# Toward Polymeric Room Temperature Acid Generators

**DOI:** 10.1002/open.202400289

**Published:** 2024-11-26

**Authors:** Joel W. Roberts, Amie N. Lanzendorf, Jazmin E. Aguilar‐Romero, Mara L. Paterson, Catherine A. Jalomo, Ephraim G. Morado, Steven C. Zimmerman

**Affiliations:** ^1^ Department of Chemistry University of Illinois at Urbana-Champaign Urbana, IL 61801 USA

**Keywords:** Acid generator, Acid amplification, Cancer, Drug delivery, Extra cellular pH

## Abstract

Although a variety of acid‐generating molecules have been developed, the formation of toxic byproducts and the need for light‐activation or temperatures that may be incompatible with physiological conditions leave room for the optimization of biocompatible acid‐generators. Herein, we report 4‐hydroxybenzyl chloride derivatives that generate hydrochloric acid via hydrolysis at the benzylic position at room temperature in the absence of light. Utilizing the acetal protected 4‐hydroxybenzyl chloride scaffold, we access a myriad of compounds that generate acid at different rates.

## Graphical abstract

A series of 4‐chloromethylphenyl acetals have been prepared and studied as thermal acid generators in water. Simple mechanistic studies show that a potential acid amplified pathway involving acetal hydrolysis and quinone methide formation is out‐competed by a simple hydrolysis pathway. The rate of thermal acid generation can be tuned through substituent effects and by incorporating the small molecule into a ROMP polymer.

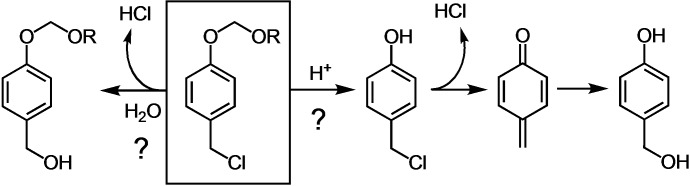

## Introduction

**Scheme 1:**
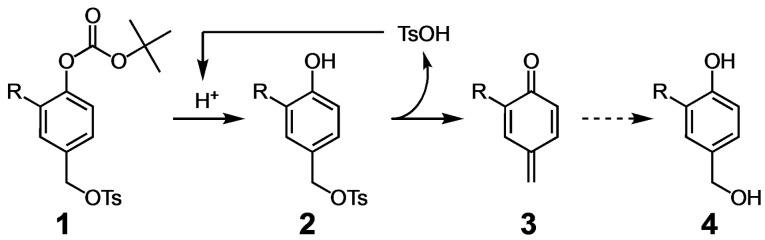
Ichimura AA **1** and its proposed mechanism of auto‐catalytic acid generation (**1**→**2**→**3**). R=H or NO_2_ and reaction occurred in acetonitrile at 70 °C. Quinone methide **3** reacts with nucleophiles such as water to produce **4**.

Photoacid generators (PAGs) are a well‐known class of stimuli‐responsive compounds that produce stoichiometric acid upon irradiation.[^1^, ^2^] PAGs have found widespread use in the development of photoresists that have proven useful in the manufacture of integrated circuits.^[3]^ To improve the performance of the photolithographic process, considerable effort has focused on a type of chemical adjuvant known as acid amplifiers (AAs). Most commonly, AAs are compounds that are stable at room temperature and neutral pH, but in the presence of acid and heat will rapidly generate additional acid in an amplified manner.[^4^, ^5^] Thus, the same amount of acid can be produced through an autocatalytic, acid‐amplifying cascade while using significantly lower concentrations of the triggering PAG. Two classes of acid amplifiers have been independently and extensively studied by the Brainard and Ichimura groups. The Brainard amplifiers are based on the 3‐hydroxypropyl sulfonate ester moiety^[6]^ whereas the Ichimura amplifier (see **1**, Scheme 1) features Boc protected 4‐hydroxy‐benzyl sulfonates.^[7]^

**Scheme 2:**
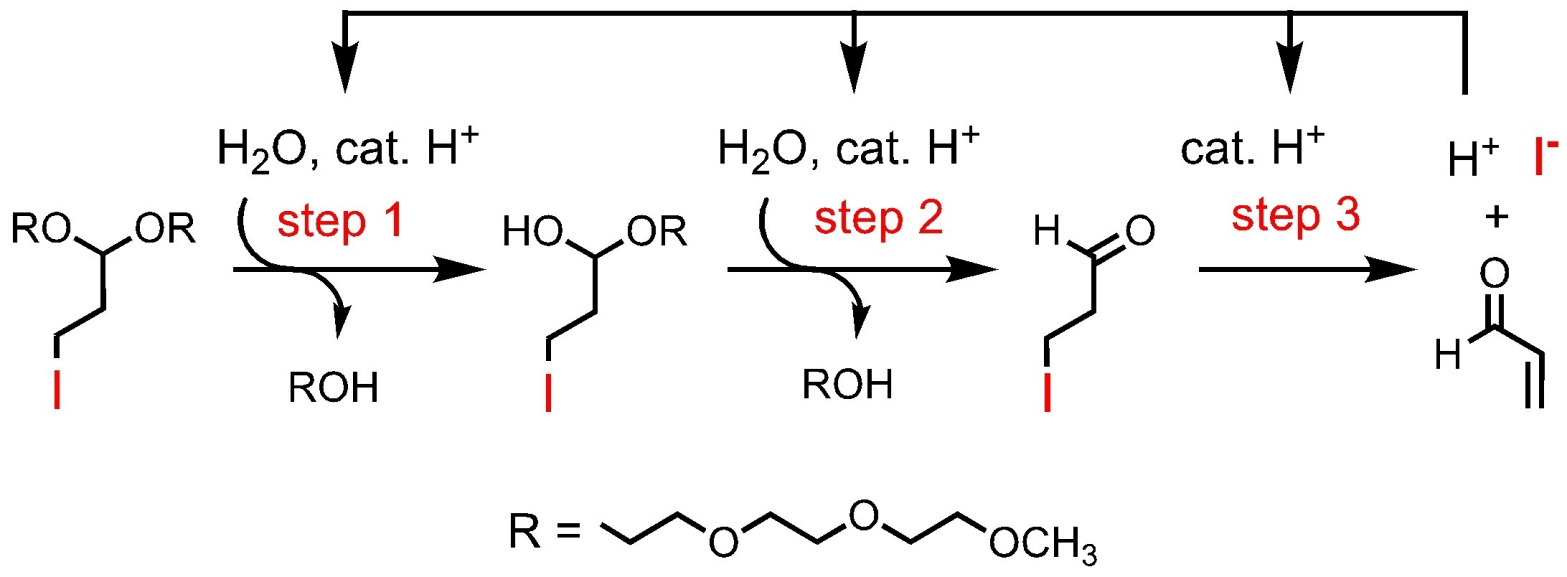
Mechanism by which 3‐iodopropyl acetal undergoes acid amplified cleavage with liberation of HI and acrolein. Analogous polyacetals underwent acid amplified chain cleavage at elevated temperatures.

We reported both a small molecule acid amplifier (Scheme 2) and an analogous polyacetal acid amplifier that demonstrated thermal chain‐shattering depolymerization in a slightly acidic aqueous‐organic medium with autocatalytic generation of the very strong acid, hydroiodic acid (HI, p*K*_a_=−10).^[8]^ Although this approach can profitably be applied to photolithography, we were interested in amplifying small pH gradients in pathological systems such as the tumor^[9]^ and bacterial film^[10]^ micro‐environments for selective drug delivery. This is a particular challenge because such applications involve sensing very small pH differences, e. g., 0.4 units below physiological pH. In this regard, our HI‐generating polymer required temperatures not compatible with living systems and initial pH values (≤pH 5) that were lower than desired.^[8]^ Furthermore, the highly toxic acrolein byproduct is inherently problematic for biological applications.

In seeking an alternative acid amplifying unit that would be responsive at physiological temperatures and only slightly acidic environments, we were attracted to the Ichimura AAs such as **1**,^[7]^ which was proposed to undergo acid catalyzed loss of the Boc protecting group forming phenol **2**, followed by formation of quinone methide **3** to produce stoichiometric *p*‐toluenesulfonic acid (TsOH). The sigmoidal decomposition is consistent with the autocatalytic formation of TsOH that can catalyze further Boc deprotection. Although the fate of quinone methide **3** was not determined in aqueous solution, it is expected to react rapidly with water, generating nontoxic gastrodigenin (**4**).^[11]^ Herein, we report the synthesis and study of a series of 4‐chloromethyl phenyl acetals designed to generate hydrochloric acid (HCl) under mild physiological conditions.

## Results and Discussion

**Scheme 3:**
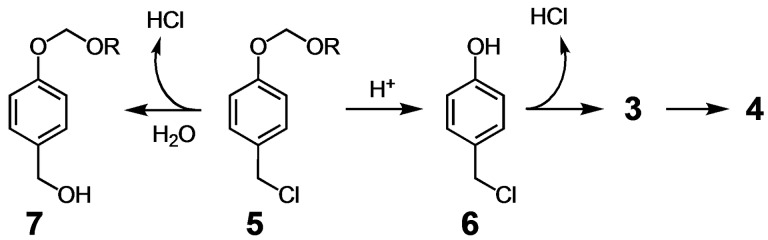
Acetal **5** studied here and two possible mechanisms for its decomposition to produce HCl.

Analogous to the acid amplifier developed by Ichimura, we designed potential acid amplifiers such as **5** with the idea that acetal hydrolysis and the subsequent quinone methide elimination might accelerate production of HCl (p*K*_a_≈−6.3), leading to amplification of an acidic stimulus. We sought to develop a compound that is stable at physiological pH=7.4, but degradable under mildly acidic conditions, e. g., pH≈6.0–7.0, such as that found in the extracellular space of tumors. Mindful of work that showed 4‐chloromethylphenols to require deprotonation to form quinone methides,^[12]^ we sought to answer several questions in this work. Would acetals such as **5** undergo amplified HCl generation by a similar mechanism proposed for **1** (see **5**→**6**→**3**→**4** in Scheme 3), or might the benzyl chloride simply undergo uncatalyzed hydrolysis to generate acid in a nonamplified, but still useful fashion (see **5**→**7**)?

**Scheme 4:**
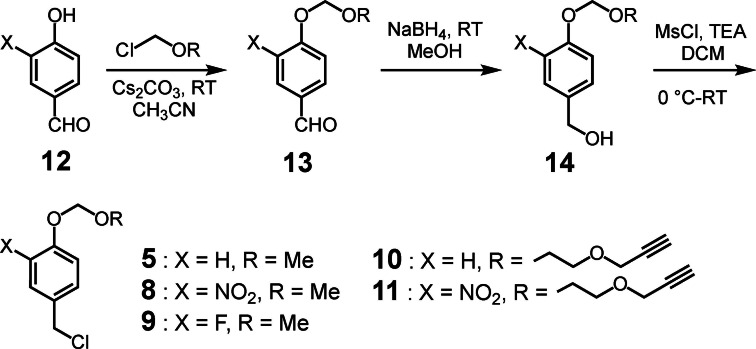
Synthetic route to acetals used in this study.

To answer these questions, benzyl chloride acetals **5** and **8**–**11** were prepared and evaluated. The substituent R^1^ was utilized to alter the rate of reaction by potentially tuning the pH sensitivity whereas the alkyne unit was introduced to provide a way to connect the unit via a click reaction. Overall, this scaffold was easily prepared, and the substituted derivatives exhibited the desired increasing stability at physiological pH (*vide infra*). The synthesis of compounds **5** and **8**–**11** is shown in Scheme 4. The synthesis of **5** was achieved by the treatment of 4‐hydroxybenzaldehyde (**12**, X=H) with MOMCl, followed by the reduction of aldehyde **13** and conversion of the resultant benzyl alcohol **14** to the corresponding benzyl chloride by treatment with methane sulfonyl chloride and triethylamine. Acetals **8** and **9** were prepared in an analogous fashion. For molecules **10** and **11**, the propargyl chloromethyl ether protecting unit was synthesized by treating 2‐(prop‐2‐ynyloxy)ethanol with TMSCl and para‐formaldehyde (see Supporting Information).

**Figure 1:**
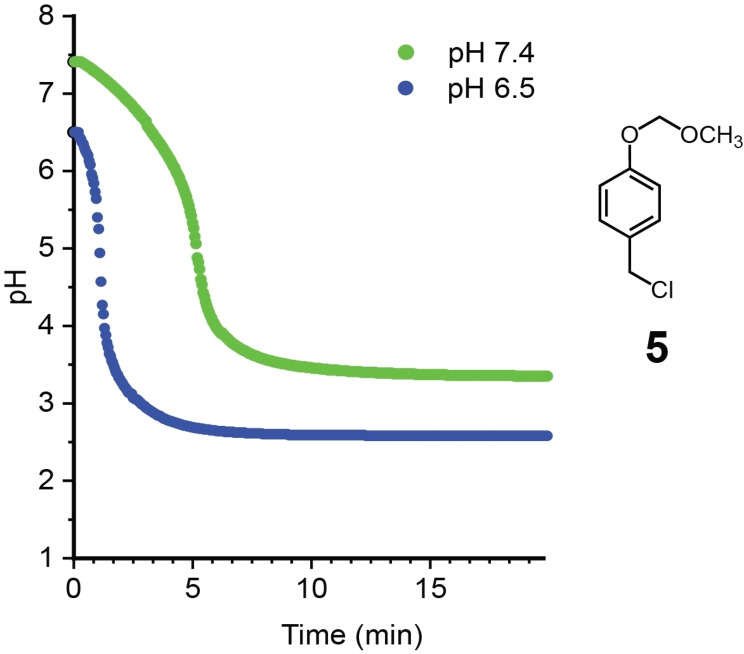
Representative pH vs time curves of 10 mM solution of **5** in 30 % (v/v) MeCN/H_2_O PBS buffer. Reported averages are based on 3 independent trials (see Figure S1). Reported error is standard error.

We initially performed pH studies with acetal **5** to test the potential generation of acid and the degree of sensitivity to pH. A solution of 30 % (v/v) MeCN/H_2_O PBS buffer was used both to mimic biological conditions and aid in the solubility of the small molecules. After the solutions were adjusted to a starting pH of 6.5 or 7.4, 10 mM of **5** was added and the pH was monitored over the course of 30 min. At a starting pH of 6.5, acid was rapidly produced, resulting in a sigmoidal curve with an inflection point at 1.33±0.20 min and reaching a final pH of 2.60±0.02 at 30 min (Figure 1). At the higher starting pH of 7.4, the pH drop was slightly slower, with the inflection point at 4.85±0.52 min, and a final pH of 3.30±0.05 due to the increased buffering capacity of the PBS solution.

**Figure 2:**
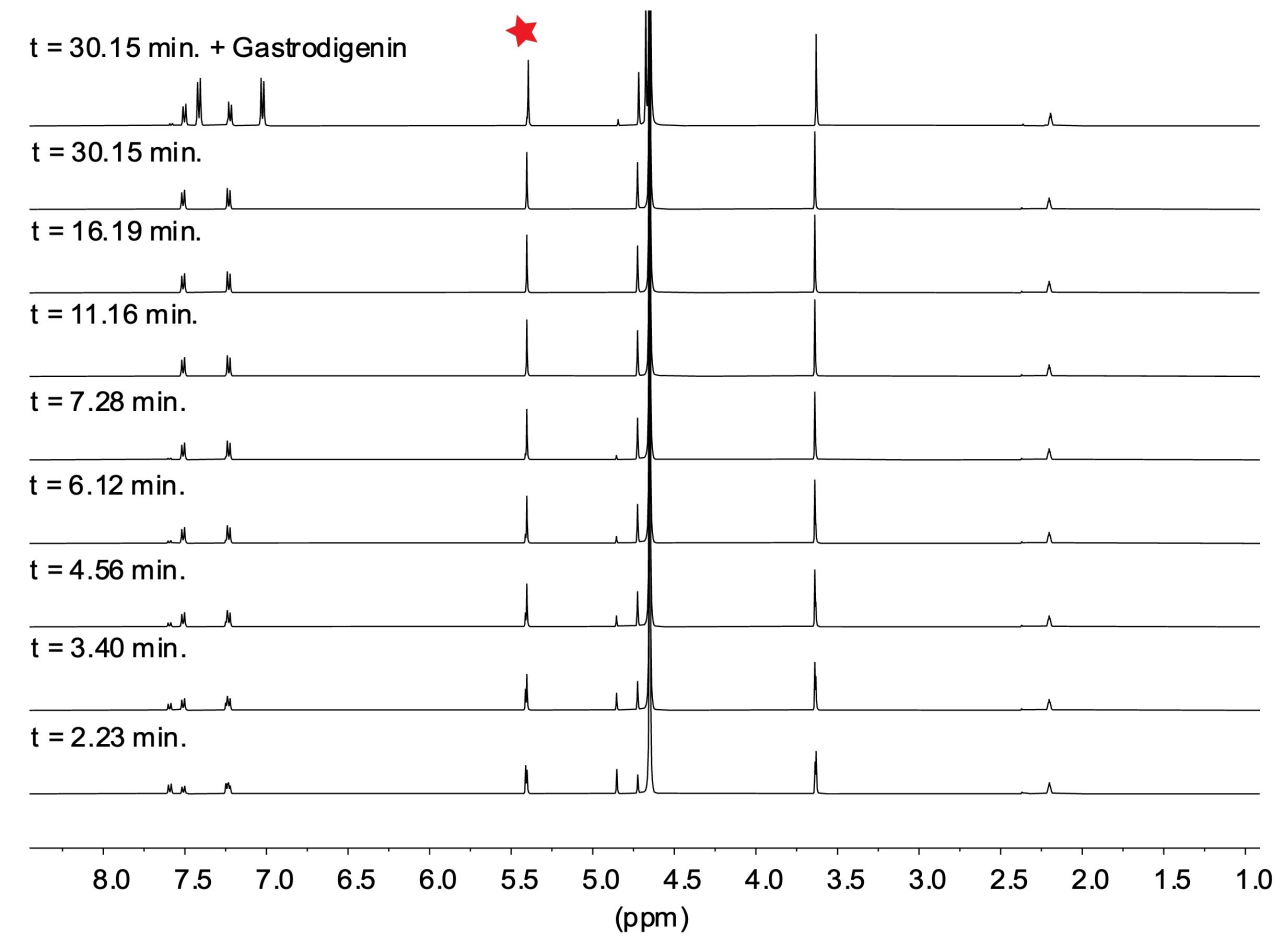
NMR spectra over time of 10 mM **5** in 30 % (v/v) MeCN‐*d*_3_/D_2_O PBS buffer (initial pD=7.4). The red star indicates the key acetal peak.

To probe the mechanism of the degradation of **5**, the reaction was monitored via ^1^H NMR using 30 % (v/v) MeCN‐*d*_3_/D_2_O PBS buffer at pD=7.4 over 30 min (Figure 2). Although the appearance of a new benzyl peak at 4.73 ppm provided evidence for the loss of the benzyl chloride, the acetal peak at 5.40 ppm remained, indicating that acetal hydrolysis was not occurring under these conditions. Furthermore, doping the reaction with gastrodigenin (**4**), the expected product after the nucleophilic attack by water on quinone methide **3**, resulted in the appearance of two new peaks at 7.02 ppm and 7.41 ppm (Figure 2), providing evidence that the quinone methide degradation pathway did not occur (Figure 2). LC–MS showed a major peak (>90 %) with *m*/*z*=168.1 that corresponds to compound **14**, and ^1^H and ^13^C NMR spectra of the reaction product obtained from HPLC purification are also fully consistent with **14** further supporting rapid hydrolysis of the benzyl chloride without acetal hydrolysis (Figures S2 and S3). The pH and NMR degradation studies indicate that the benzyl chloride moiety of **5** is not stable at physiological pH, rapidly generating acid by hydrolysis without acetal cleavage. These results are consistent with the water‐mediated **5**→**7** conversion shown in Scheme 3.

We next sought to make derivatives of **5** that would stabilize the benzyl halide moiety, allowing the rate of the spontaneous hydrolysis and acid generation to be tuned. This rate reduction was accomplished by adding an electron‐withdrawing nitro‐ or fluoro‐group ortho to the acetal group (see **8** and **9**, respectively).^[13]^ Additionally, these electron‐withdrawing groups (EWGs) lower the p*K*_a_ of the resulting phenol, which could potentially allow quinone methide formation to occur more readily if acetal hydrolysis preceded benzyl chloride hydrolysis.^[12]^ To provide a means for linking these acid‐generating compounds to other molecular entities we synthesized derivatives (**10** and **11**) containing propargyl ether groups. Although propargyl alcohol itself could have been used, we chose to use 2‐propynoxyethanol based on reports that 4‐alkoxy groups can increase the rate of acetal hydrolysis by up to 200‐fold.^[14]^

**Figure 3:**
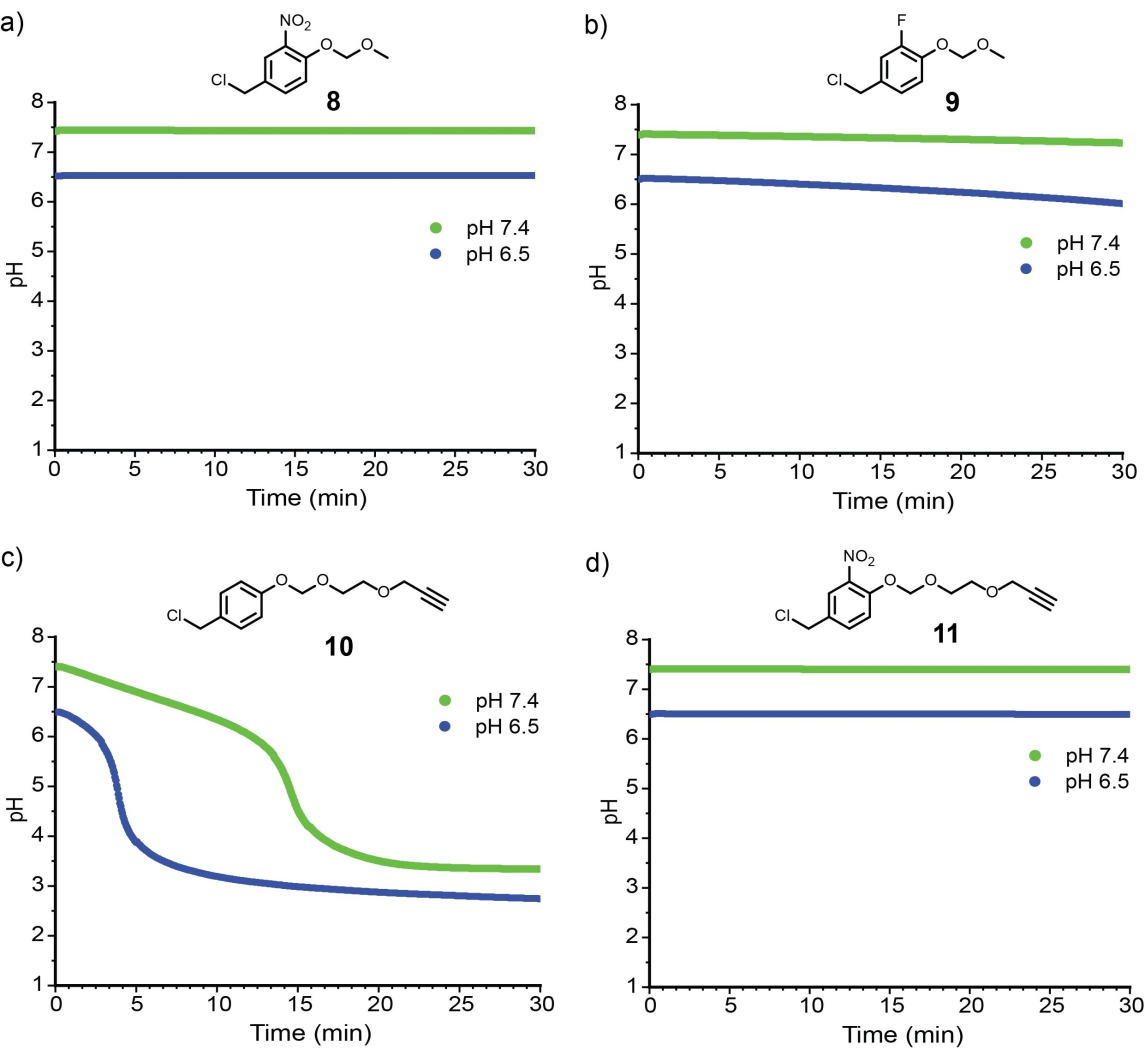
Representative pH vs time curves of (a) **8** (b) **9**, (c) **10**, and (d) **11**. All reactions were conducted with an initial concentration of 10 mM compound in 30 % (v/v) MeCN/H_2_O PBS buffer. Reported averages are based on 3 independent trials (Figures S4, S5, S6, and S7). Reported errors are standard error.

In the pH studies, the benzyl chlorides of **8** and **9** showed increased stability with no drop in pH at 7.4 for either compound and only a slight drop from 6.5–6.16±0.15 after 30 min for **9** (Figures 3a and b). The electron‐withdrawing groups were not expected to have a significant effect on acetal hydrolysis, and indeed ^1^H NMR studies confirmed that no acetal hydrolysis was observed over 20 and 30 min for **8** and **9**, respectively (Figures S8 and S9). Despite the possibility that the alkoxy group in compound **10** could increase the rate of acetal hydrolysis,^[14]^ NMR studies indicated that the acetal remained intact with the peak at 5.46 ppm remaining unchanged (Figure S10). Nonetheless, as with **5**, benzyl chloride hydrolysis occurred with the benzylic peak shifting from 4.84 ppm–4.71 ppm. The pH studies on **10** showed a drop in pH over 30 min that was noticeably slower in comparison to **5** (Figure 3c). At a starting pH of 6.5, the inflection point of the sigmoidal curve occurred at 3.2±0.6 min and reached a final pH of 2.73±0.12. At a starting pH of 7.4, the inflection point occurred at 14.4±2.8 min and reached a final pH of 3.31±0.06 (Figure 3a). Although we did not anticipate that the alkynyl‐containing group in **10** would lead to any significant difference in the benzyl chloride hydrolysis rates when compared to **5**, this result demonstrates that even distant groups may influence the rate of hydrolysis.

Compound **11** contains both an electron‐withdrawing nitro group on the ring as well as a propargyl ether group next to the acetal. Interestingly, although there was no drop in pH over 30 min (Figure 3d), the NMR studies showed a shift in all of the peaks including the acetal peak (Figure S11). However, the acetal peak shifted to 5.61 ppm, which is still within the acetal region, suggesting that the acetal had not undergone hydrolysis. At this time, the mechanism of degradation for **11** is unknown. The aromatic substituents in compounds **8**, **9**, and **11** effectively slowed their rates of acid generation, but for the applications of interest these compounds were too stable to hydrolyze under physiologically relevant conditions.

Although compounds **5** and **8**–**11** did not undergo autocatalytic acid generation, **5** produced acid with significant drops in pH under slightly acidic conditions. Thus, it has clear potential to amplify mildly acidic biological conditions. Toward our ultimate goal to use these acid generators in polymeric drug delivery systems for cancer, we sought preliminary evidence that analogs of **5** could be integrated into a polymer and still function as acid generators. Beyond its potential to target tumor tissue using the enhanced permeability and retention (EPR) effect,^[15]^ the water‐soluble polymer was expected to fold hydrophobically, thereby protecting the chloromethyl groups from alkylating proteins and other nucleophilic biomacro‐molecules. To this end, norbornene monomer (**17**) was synthesized with acid‐generating side chains based on **5** to enable ring opening metathesis polymerization (ROMP). A hydrophilic comonomer (**18**) was also synthesized using Jeffamine M‐1000 to enable water solubility for pH studies.

**Scheme 5:**
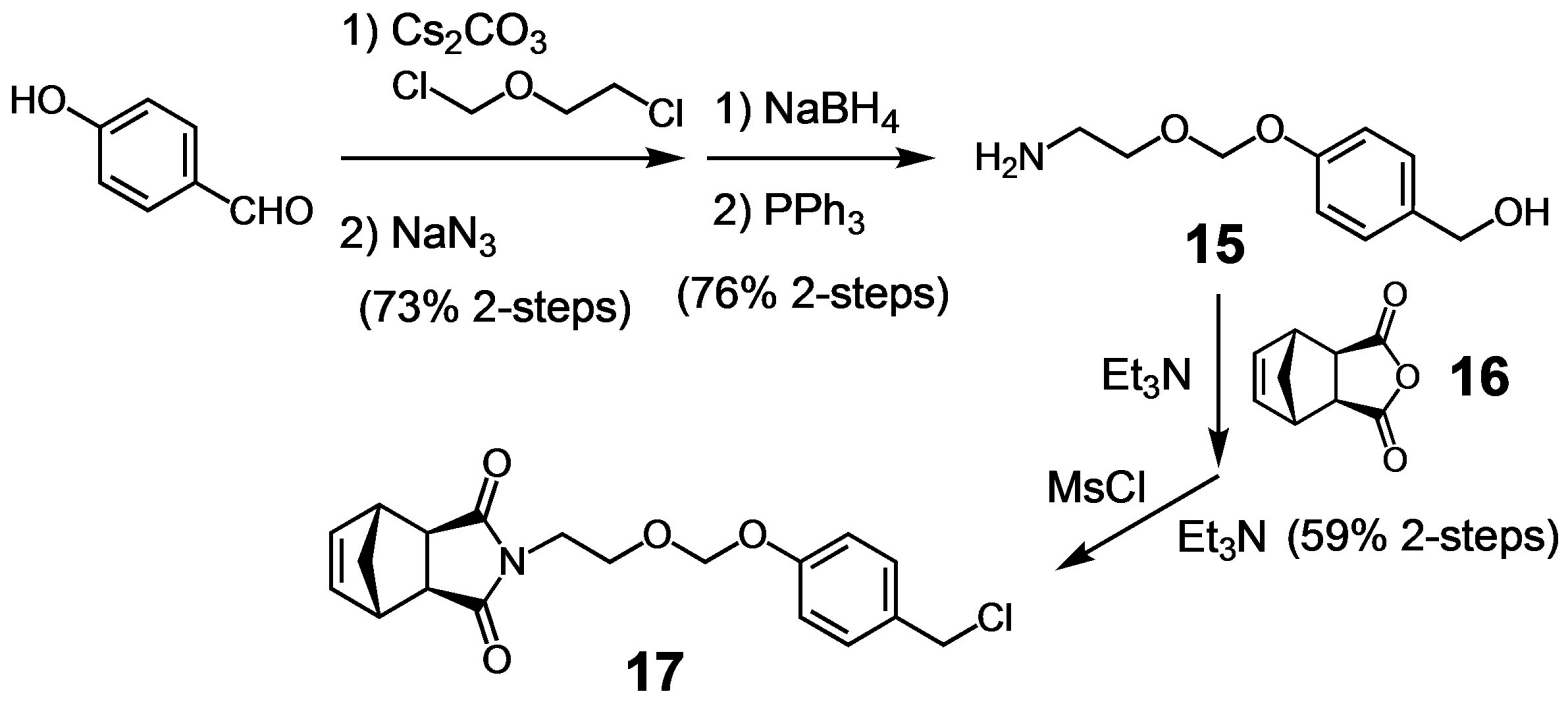
Synthesis of acid generating monomer **17**.

**Scheme 6:**
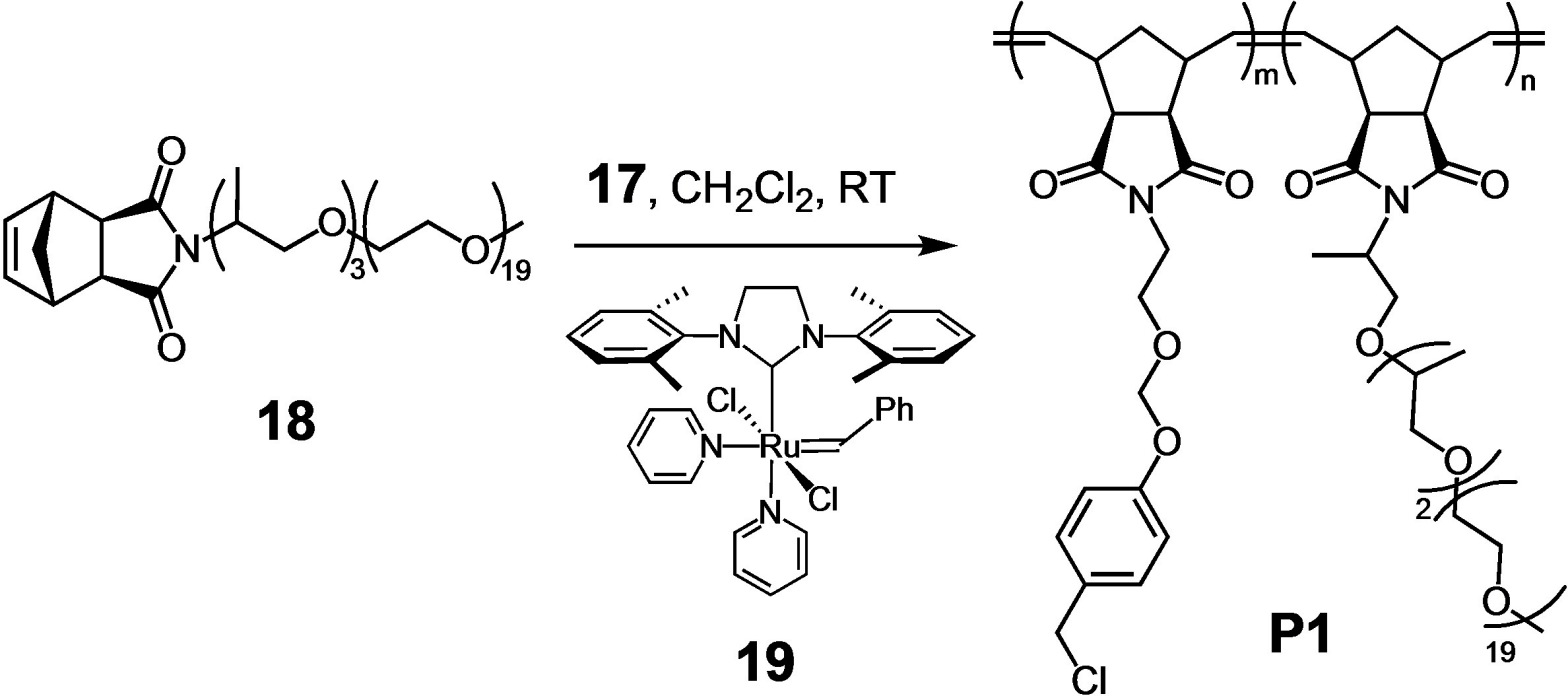
Synthesis of **P1** by ROMP using Grubbs catalyst **19**.

A polynorbornene copolymer was prepared with a 1 : 1 ratio of monomers **17** and **18** (Scheme 5). Based on the polymerization procedure and previously reported syntheses of ROMP polymers from similar norbornene imide monomers, statistical copolymers would form (see Scheme 6).[^16^, ^17^] Using size exclusion chromatography (SEC) with THF as solvent, **P1** was shown to have *M_n_* =138 kDa and *Ð*=1.3. The ratio of acid generator to Jeffamine monomer was determined using ^1^H NMR based on characteristic acetal proton peaks for the acid generator (δ 5.17) and the terminal methyl peaks (δ 3.38) for Jeffamine M‐1000 (Figure S12). Based on this calculation, **P1** was composed of ca. 43 % acid generator and 57 % Jeffamine monomer.

**Figure 4:**
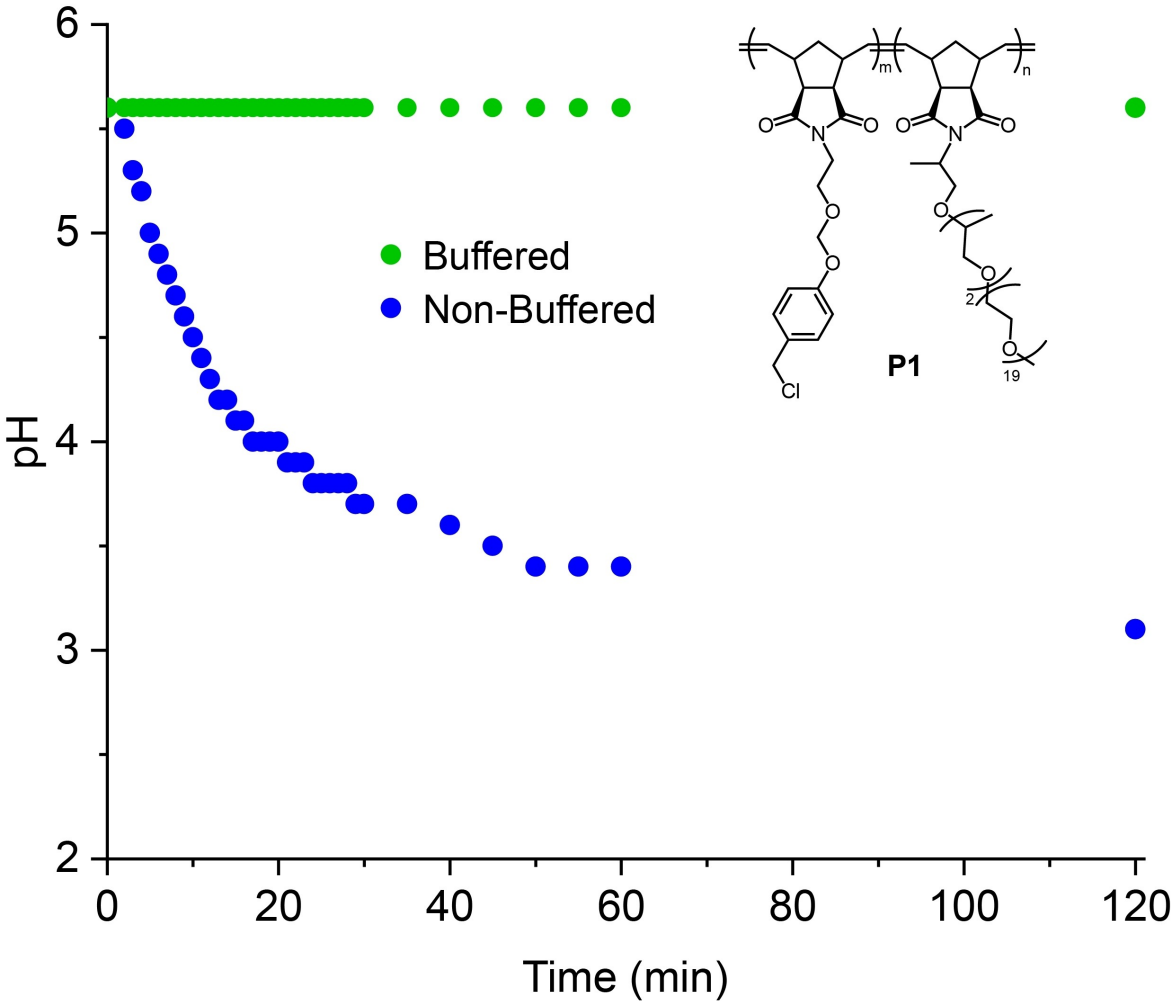
Plot of pH over time with 5 mg/mL of **P1** in deionized water initial pH=5.6 (blue) and 0.1 M acetate buffer initial pH=5.6 (green).

To determine if **P1** was indeed acid‐generating, the pH of solutions containing 5 mg/mL of **P1** were monitored. To achieve a suitable reaction rate at 25 °C, an initial pH of 5.6 was required. In non‐buffered conditions, the pH drop was observed almost immediately with **P1**, reaching pH 5.0 in 5 min and pH 3.7 after 30 min. (Figure 4). Degradation studies in pH 5.6 with 0.1 M acetate buffer were conducted as a negative control and demonstrated no drop in pH after 30 min, suggesting that either no acid was generated, or the buffer was able to maintain the initial pH.

## Conclusions

We have investigated how MOM‐protected 4‐hydroxybenzyl chloride acid‐generating molecules behave at physiological and pathological pH. Compound **5** was shown to generate acid rapidly via hydrolysis at the benzylic position. Mechanistic studies showed that the acid generation does not require acetal hydrolysis and thus does not occur by an acid‐amplifying mechanism. Based on the effects that electron‐withdrawing groups have on benzylic leaving group ability, it was not surprising that adding a nitro‐ or fluoro‐group meta to the benzylic position stabilized **8**, **9** and **11** against hydrolysis. It was interesting, however, that the propargyl group on **10** increased its stability compared to **5**, showing that remote electron‐withdrawing groups can affect the reaction kinetics, although via an unknown mechanism at this time.

A ROMP copolymer containing unit **5** was prepared and found to be soluble in mixed aqueous‐organic solvent and similarly found to generate acid albeit at a slower rate and requiring a lower starting pH, which is below that found in the extracellular space of tumors. Further efforts will be needed to optimize the properties of these molecules so that they might generate acid and amplify the slightly lower pH values found in pathological conditions such as seen in cancer and bacterial infections. Additionally, whether the polymer can encapsulate the chloromethyl groups allowing a suitable degree of biocompatibility will require testing and optimization. The ability to amplify the slightly acidic pH within tumors could be immensely useful for enhancing selectivity of covalent drug release while at the same time potentially allowing the polymer to degrade or otherwise alter its size. Although large polymers accumulate selectively within tumors, small sizes are better able to penetrate deeply.

## Experimental Section

Dry solvents were stored under activated 4 Å molecular sieves. All reactions were run under N_2_ atmosphere with oven‐dried glassware. Column chromatography was performed on silica gel (60 Å pore size, 230–400 mesh). NMR spectra were recorded using Varian UI500NB or Bruker CB500 or B600 spectrometers. NMR spectra were processed using MestReNova software and chemical shifts reported in ppm. All ^1^H spectra were referenced to the residual solvent peak. Integration is provided and coupling constants (*J*) are reported in Hertz (Hz). Mass spectrometry was performed using a Waters Q‐TOF Ultima ESI. LC–MS was performed using a Waters Synapt G2‐Si. Acid generation studies were conducted using a Thermo Scientific Orion Star A221 pH meter, and values were recorded with accompanying Star Com software. Results were analyzed using Origin 2023 software. HPLC was performed on Agilent Technologies 1260 Infinity II, C‐18 250×50 mm column.

### Representative Procedure for Preparation of Benzyl Chloride Acetals

To a suspension of the benzaldehyde (1 equivalent) and cesium carbonate (2 equivalents) in anhydrous acetonitrile, the chloro‐(methoxy)alkane was added at room temperature and stirred for 12 h. The suspension was concentrated using a rotary evaporator, washed with water (3x) and brine (1x), and the organic layer was dried with anhydrous sodium sulfate, filtered and concentrated using a rotary evaporator. The crude product was redissolved in anhydrous methanol, and sodium borohydride (2 equivalents) was added, and the mixture stirred at room temperature for 2 h, monitoring by TLC (hexanes:EtOAc). The reaction mixture was quenched with the addition of water, concentrated under reduced pressure, and redissolved in EtOAc. The solution was washed with water (3x) and brine (1x), and the organic layer was dried with anhydrous sodium sulfate, filtered and concentrated by rotary evaporation. The crude product was dissolved in anhydrous dichloromethane and 2 equivalents of triethylamine and methane sulfonyl chloride added sequentially at 0 °C. The solution was warmed to room temperature and stirred, monitoring reaction completion by TLC (hexanes:EtOAc). The mixture was diluted with DCM and washed three times with a 10 % (w/w) aqueous sodium bicarbonate solution. The organic layer was dried with sodium sulfate, filtered, and concentrated using a rotary evaporator. The residue was purified via flash chromatography on silica (hexanes:EtOAc) to afford the pure benzyl chloride acetal. Yields and characterization data (NMR and HR‐ESI‐MS) for **5** and **8**–**11** can be found in the Supporting Information.

## Conflict of Interests

The authors declare no conflict of interest.

1

## Supporting information

As a service to our authors and readers, this journal provides supporting information supplied by the authors. Such materials are peer reviewed and may be re‐organized for online delivery, but are not copy‐edited or typeset. Technical support issues arising from supporting information (other than missing files) should be addressed to the authors.

Supporting Information

## Data Availability

The data that support the findings of this study are available in the supplementary material of this article.
