# Supplementary material for: Toward Polymeric Room Temperature Acid Generators
Source: ChemistryOpen. 2024 Nov 26;14(3):e202400289. doi: 10.1002/open.202400289 (PMC11891450; doi:10.1002/open.202400289)
Supplement: Supplementary file 1 — Supporting Information [file OPEN-14-e202400289-s001.pdf]

ChemistryOpen

Supporting Information

Toward Polymeric Room Temperature Acid Generators

Joel W. Roberts, Amie N. Lanzendorf, Jazmin E. Aguilar-Romero, Mara L. Paterson, Catherine A. Jalomo, Ephraim G. Morado, and Steven C. Zimmerman*

Supporting Information for

Toward Polymeric Room Temperature Acid Generators

Joel W. Roberts,+[a] Amie N. Lanzendorf,+[a] Jazmin E. Aguilar-Romero,+[a] Mara L. Paterson,[a] Catherine A. Jalomo,[a] Ephraim G. Morado,[a] and Steven C. Zimmerman*[a]

[a] Joel W. Roberts, Amie N. Lanzendorf, Jazmin E. Aguilar-Romero, Mara L. Paterson, Catherine A. Jalomo, Ephraim G. Morado, Department of Chemistry, University of Illinois at Urbana-Champaign, Urbana, IL, 61801, USA

+These authors contributed equally.

Table of Contents

General information S2

Synthetic procedures / compound characterization S2

ROMP copolymerization of **17** and **18** S4

pH Studies of acid generation by **5** and **8-11** S4

NMR studies of acid generation by **5** and **8-11** S4

LC MS and HPLC S5

^{1}H NMR spectra of **5** and **8-11** S5

Additional supporting figures S8

References S14

General Information. All reagents and solvents were purchased from Sigma-Aldrich, TCI America, Acros Organics, or Fisher Scientific and were used without further purification unless otherwise stated. Dry DCM and THF for reactions were obtained from a MBRAUN solvent purification system. Third-generation Grubbs catalyst was prepared based on a procedure published by Grubbs and coworkers.[1,2] Dry solvents were stored under activated 4 Å molecular sieves. All reactions were run under N_2 atmosphere with oven-dried glassware. Flash column chromatography was performed on silica gel (60 Å pore size, 230-400 mesh). NMR spectra were recorded using Varian UI500NB or Bruker CB500 or B600 spectrometers. NMR spectra were processed using MestReNova software and chemical shifts (δ) reported in ppm. All ^{1}H spectra were referenced to the residual solvent peak. Integration is provided and coupling constants (J) are reported in Hertz (Hz). Mass spectrometry was performed using a Waters Q-TOF Ultima ESI. LC-MS was performed using a Waters Synapt G2-Si. Acid generation studies were conducted using a Thermo Scientific Orion Star A221 pH meter, and values were recorded with accompanying Star Com software. Results were analyzed using Origin 2023 software. HPLC was performed on Agilent Technologies 1260 Infinity II, C-18 250 x 50.0 mm column.

Synthetic Methods and Compound Characterization. Synthetic scheme and general procedure for preparing **5** and **8-11** can be found in the manuscript. Purity and characterization data are provided here as are full spectra. The preparation of **10** and **11** required 3-(2-(chloromethoxy)ethoxy)prop-1-yne, whose preparation is described below:

Synthesis of 3-(2-(chloromethoxy)ethoxy)prop-1-yne. 2-(prop-2-yn-1-yloxy)ethan-1-ol (2.00g g, 18.0 mmol) was added to a suspension of paraformaldehyde (810 mg, 27.0 mmol) in TMSCl (9.13 mL, 71.9 mmol) at room temperature under argon. The resulting suspension was stirred until a clear solution was achieved (ca. 2 h). The solution was concentrated under reduced pressure to afford the crude oil and used in the preparation of **10** and **11** without purification.

1-(Chloromethyl)-4-(methoxymethoxy)benzene (**5**): Using the general procedure, **5** was obtained as a clear liquid (61% yield). ^{1}H (600 MHz, $CDCl_3$) δ 7.32 (d, *J* = 8.7, 2H), 7.03 (d, *J* = 8.7, 2H), 5.18 (s, 2H), 4.57 (s, 2H), 3.48 (s, 3H). ^{13}C (151 MHz, $CDCl_3$) δ 157.33, 130.93, 130.06, 116.44, 94.36, 56.05, 46.17. HR-ESI-MS: *m*/*z* calculated for $C_9H_{12}ClO_2$ (M^+): 186.045; found: 186.044.

4-(Chloromethyl)-1-(methoxymethoxy)-2-nitrobenzene (**8**): Using the general procedure, **8** was obtained as a yellow solid (14% yield): ^{1}H (600 MHz, CD_3Cl) δ 7.85 (d, *J* = 2.3, 1H), 7.53 (dd, *J* = 8.7, 2.3, 1H), 7.31 (d, *J* = 8.7, 1H), 5.30 (s, 2H), 4.56 (s, 2H), 3.52 (s, 3H). ^{13}C (151 MHz, CD_3Cl) δ 150.29, 140.41, 133.97, 131.16, 125.49, 117.48, 95.27, 64.61, 56.81, 44.47. HR-EI-MS: *m*/*z* calculated for $C_9H_{10}ClNO_4$ $(M)^+$: 231.0298; found: 231.0304.

4-(Chloromethyl)-2-fluoro-1-(methoxymethoxy)benzene (9): Using the general procedure, **9** was obtained as a clear liquid (68% yield): ^{1}H (600 MHz, CD_3Cl) δ 7.18–7.12 (m, 2H), 7.07 (dt, *J* = 8.2, 1.5, 1H), 5.22 (s, 2H), 4.52 (s, 2H), 3.52 (s, 3H). ^{13}C (151 MHz, CD_3Cl) δ 152.87 (d, *J* = 247), 145.09 (d, *J* = 10.8), 132.05 (d, *J* = 6.5), 124.62 (d, *J* = 3.4), 117.86 (d, *J* = 1.7), 116.76 (d, *J* = 19.4), 95.59, 56.42, 45.40. ^{19}F (470 MHz, CD_3Cl): -132.68 (dd, *J* = 11.5, 8.4).

1-(Chloromethyl)-4-((2-(prop-2-yn-1-yloxy)ethoxy)methoxy)-benzene (**10**): Using the general procedure, **10** was obtained as a white solid (32% yield): ^{1}H (600 MHz, CD_3Cl): 7.33–7.28 (m, 2H), 7.05–7.01 (m, 2H), 5.27 (s, 2H), 4.56 (s, 2H), 4.18 (d, *J* = 2.4, 2H), 3.87–3.83 (m, 2H), 3.72–3.68 (m, 2H), 2.43 (t, *J* = 2.4, 1H). ^{13}C (151 MHz, CD_3Cl) δ 157.32, 130.95, 130.05, 116.44, 93.37, 79.41, 74.72, 68.81, 67.61, 58.42, 46.16. HR-EI-MS: *m*/*z* calculated for $C_{13}H_{15}ClO_3$ ($M+H^+$) 254.0710; found: 254.0715

4-(Chloromethyl)-2-nitro-1-((2-(prop-2-yn-1-yloxy)ethoxy)methoxy)benzene (11): Using the general procedure, **11** was obtained as yellow solid (69% yield): ^{1}H (600 MHz, CD_3Cl) δ 7.84 (d, *J* = 2.3, 1H), 7.53 (dd, *J* = 8.7, 2.3, 1H), 7.36 (d, *J* = 8.6, 1H), 5.39 (s, 2H), 4.56 (s, 2H), 4.17 (d, *J* = 2.3, 2H), 3.93–3.88 (m, 2H), 3.73–3.66 (m, 2H), 2.43 (t, *J* = 2.4, 1H). ^{13}C (151 MHz, CD_3Cl) δ 150.28, 140.40, 134.02, 131.32,

125.47, 117.63, 94.23, 79.25, 74.83, 68.61, 68.44, 58.41, 44.47. HR-ESI-MS: *m*/*z* calculated for $C_{13}H_{14}ClNNaO_5$ (M+Na$^+$) 322.0458; found: 322.0459.

4-((2-Chloroethoxy)methoxy)benzaldehyde. A suspension containing 2.00 g (16.4 mmol) of 4-hydroxybenzaldehyde, 50 mL of acetonitrile, and 5.88 g (18.1 mmol) of cesium carbonate was prepared and stirred. The mixture was cooled in an ice bath and 1.82 mL (18.1 mmol) of 1-chloro-2-(chloromethoxy)ethane was added dropwise. The suspension was stirred for 3.5 h at room temperature. To the mixture was added 50 mL of DI water and in a separatory funnel the solution was washed three times with ethyl acetate. The organic layers were combined and dried with sodium sulfate. The solution was filtered and dried *in vacuo*, revealing a brown liquid. The crude product was purified on a silica column using an eluent gradient from 3% to 3.5% (v/v) ethyl acetate:hexanes to afford 2.44 g (69%) of the product as a yellow liquid. ^{1}H NMR (600 MHz, CDCl3) δ 9.93 (s, 1H), 7.90–7.84 (m, 2H), 7.21–7.17 (m, 2H), 5.38 (s, 2H), 3.97 (t, *J* = 5.7, 2H), 3.66 (t, *J* = 5.7, 2H). ^{13}C NMR (151 MHz, $CDCl_3$) δ 191.01, 162.11, 132.03, 131.08, 116.45, 93.17, 69.26, 42.78. HR-ESI-MS *m/z* calculated for $C_{10}H_{12}ClO_3$ (M+H$^+$): 215.047; found: 215.048.

4-((2-Azidoethoxy)methoxy)benzaldehyde. A suspension of 2.40 g (11.2 mmol) of 4-((2-chloroethoxy)-methoxy)benzaldehyde, 1.09 g (16.8 mmol) of sodium azide, and 30 mL of DMF was prepared and stirred at 60 °C overnight. To the mixture was 30 mL of ethyl acetate and in a separatory funnel the solution was washed three times with 15 mL DI water and once with 15 mL of brine. The organic layer was dried with sodium sulfate, filtered, and dried *in vacuo*. The crude product was purified on a silica column using 10% (v/v) ethyl acetate:hexanes, affording 2.00 g (81%) of the product as a yellow liquid. ^{1}H NMR (600 MHz, CDCl3) δ 9.91 (d, *J* = 1.5, 1H), 7.88–7.82 (m, 2H), 7.19–7.14 (m, 2H), 5.35 (s, 1H), 3.85 (t, *J* = 5.1, 2H), 3.41 (t, *J* = 5.0, 2H). ^{13}C NMR (151 MHz, $CDCl_3$) δ 191.03, 162.05, 132.07, 131.07, 116.38, 93.07, 67.96, 50.82. HR-ESI-MS: *m/z* calculated for $C_{10}H_{11}N_3O_3$ (M$^+$): 221.080; found: 221.080.

(4-((2-Azidoethoxy)methoxy)phenyl)methanol. A solution of 2.00 g (9.04 mmol) of 4-((2-azidoethoxy)methoxy)benzaldehyde, 30 mL of methanol, and 103 mg (2.71 mmol) of sodium borohydride was stirred at room temperature under nitrogen for 1 h. To the mixture was added 30 mL of DI water and the solution was washed three times in a separatory funnel with 90 mL of ethyl acetate. The organic layers were collected, dried with sodium sulfate, and filtered. The solution was dried *in vacuo*, revealing a clear liquid. The crude product was purified on a silica column using an eluent gradient of 10% to 25% (v/v) ethyl acetate:hexanes to afford 1.16 g (58%) the product as a clear liquid. 1H NMR (600 MHz, $CDCl_3$) δ 7.32–7.28 (m, 2H), 7.06–7.02 (m, 2H), 5.27 (s, 2H), 4.63 (d, *J* = 5.1, 2H), 3.84 (dd, *J* = 5.6, 4.6, 2H), 3.41 (t, *J* = 5.1, 2H). ^{13}C NMR (151 MHz, $CDCl_3$) δ 156.77, 134.70, 128.76, 116.38, 93.45, 67.49, 65.12, 50.91. HR-ESI-MS: *m/z* calculated for $C_{10}H_{13}NaN_3O_3$ (M+Na$^+$): 246.085; found: 246.085.

(4-((2-Aminoethoxy)methoxy)phenyl)methanol. A solution of 716 mg (3.21 mmol) of (4-((2-azidoethoxy)-methoxy)phenyl)methanol, 10 mL of dry THF, and 925 mg (3.53 mmol) of triphenylphosphine was prepared and stirred at room temperature overnight. The mixture was quenched with 10 mL of DI water and stirred for an additional hour. To the solution was added 1 M HCl to lower the pH to 6.0. The aqueous layer was ten times in a separatory funnel with 10 mL ethyl acetate, occasionally checking the organic layers via TLC for any triphenylphosphine oxide. The aqueous layer was dried *in vacuo* to afford 527 mg (87%) of the product as a clear liquid. ^{1}H NMR (600 MHz, $CDCl_3$) δ 7.24 (d, *J* = 8.3, 2H), 6.98 (d, *J* = 8.3, 2H), 5.17 (s, 2H), 4.56 (s, 2H), 3.70 (t, *J* = 5.2, 2H), 2.73 (t, *J* = 5.2, 2H). ^{13}C NMR (151 MHz, $CDCl_3$) δ 156.76, 134.72, 128.66, 116.33, 93.54, 67.95, 64.82, 48.89. HR-ESI-MS: *m/z* calculated for $C_{10}H_{16}NO_3$ (M+H$^+$): 198.112; found: 198.113.

(3a*R*,4*R*,7*S*,7a*S*)-2-(2-((4-(Hydroxymethyl)phenoxy)methoxy)ethyl)-3a,4,7,7a-tetrahydro-1*H*-4,7-methanoisoindole-1,3(2*H*)-dione. A suspension of 633 mg (3.21 mmol) of (4-((2-aminoethoxy)methoxy)-phenyl)methanol, 632 mg (3.85 mmol) of cis-norbornene-exo-2,3-dicarboxylic anhydride (himic acid)**,** 20 mL of toluene, and 44.7 µL (321 µmol) of triethylamine was prepared. A Dean Stark apparatus was set up and the flask was heated gradually to 125 ºC while stirring the suspension under nitrogen gas overnight. The flask was removed from heat and 15 mL of DI water were added to the mixture. The aqueous layer was washed three times in a separatory funnel with 20 mL ethyl acetate and the organic layers were collected, washed with brine, dried with sodium sulfate, and filtered. The solvent was removed by rotary evaporation to afford an orange liquid. The crude product was purified on a silica column using a gradient

of 25% to 50% (v/v) ethyl acetate:hexanes to afford 0.980 g (89%) of the product as a clear liquid. ^{1}H NMR (600 MHz, $CDCl_3$) δ 7.25 (d, *J* = 9.0, 2H), 6.95 (d, *J* = 7.7, 2H), 6.23 (t, *J* = 1.8, 2H), 5.16 (d, *J* = 1.6, 2H), 4.60–4.57 (m, 2H), 3.83 (t, *J* = 5.5, 2H), 3.69 (t, *J* = 5.5, 2H), 3.20–3.16 (m, 2H), 2.46 (d, *J* = 1.7, 2H), 1.36 (d, *J* = 10.0, 1H), 1.18 (d, *J* = 9.8, 1H). ^{13}C NMR (151 MHz, CDCl3) δ 178.07, 156.70, 137.88, 134.57, 128.67, 116.29, 92.49, 65.01, 64.41, 47.88, 45.25, 42.78, 38.13. HR-ESI-MS: *m/z* calculated for $C_{19}H_{21}NaNO_5$ ($M+Na^+$): 366.131; found: 366.131.

(3aR,4R,7S,7aS)-2-(2-((4-(Chloromethyl)phenoxy)methoxy)ethyl)-3a,4,7,7a-tetrahydro-1H-4,7-methanoisoindole-1,3(2H)-dione (17). A solution of 232 mg (0.68 mmol) of (3a*R*,4*R*,7*S*,7a*S*)-2-(2-((4-(hydroxymethyl)phenoxy)methoxy)ethyl)-3a,4,7,7a-tetrahydro-1*H*-4,7-methanoisoindole-1,3(2*H*)-dione, 0.15 mL (1.08 mmol) of TEA, and 2 mL of dichloromethane was prepared and stirred at room temperature for 20 min. The mixture was cooled in an ice bath and 60 µL (0.74 mmol) of MsCl was added dropwise. The solution was stirred at room temperature for 2 h. The solution was placed in an ice bath and to the mixture was added 2 mL of cooled saturated sodium bicarbonate. The aqueous layer was washed three times with 2 mL of dichloromethane. The organic layers were collected, dried with sodium sulfate, filtered, and the solvent removed by rotary evaporation to afford a white solid. The crude product was purified on a silica column using a gradient of 15% (v/v) ethyl acetate:hexanes to 100% ethyl acetate with 0.5% (v/v) TEA to afford 137 mg (56%) of the product as a white solid: ^{1}H NMR (600 MHz, $CDCl_3$) δ 7.30–7.27 (m, 2H), 6.96–6.93 (m, 2H), 6.22 (s, 2H), 5.17 (s, 2H), 3.85 (t, *J* = 5.4, 2H), 3.70 (t, *J* = 5.4, 2H), 3.17 (t, *J* = 1.8, 2H), 2.43 (s, 1H), 1.34 (d, *J* = 9.9, 1H), 1.14 (d, *J* = 10.0, 1H). ^{13}C NMR (151 MHz, $CDCl_3$) δ 178.13, 157.13, 137.81, 131.10, 130.20, 116.36, 92.20, 64.53, 47.86, 46.19, 45.31, 42.76, 38.18. HR-ESI-MS: *m/z* calculated for $C_{19}H_{22}ClO_5$ ($M+H^+$): 362.115; found: 362.115.

Jeffamine M-1000 norbornene monomer (18). A suspension of 18.5 g (17.79 mmol) of Jeffamine 1000, 2.78 g (16.94 mmol) of cis-norbornene-exo-2,3-dicarboxylic anhydride**,** 100 mL of toluene, and 2.60 mL (1.86 mmol) of triethylamine was prepared. A Dean Stark apparatus was set up and the flask was heated gradually to 125 ºC while stirring the suspension under nitrogen gas overnight. The flask was removed from heat and the solution was dried *in vacuo*. The crude product was dissolved in 100 mL dichloromethane and washed with 20 mL 0.1 M HCl and 20 mL of brine. The organic layer was dried with sodium sulfate, filtered, and dried *in vacuo* to afford an off-white viscous oil. The crude product was purified on a silica column using a gradient of 5% to 20% (v/v) methanol:dichloromethane to afford 6.94 g (35%) of the product as a white cream. ^{1}H NMR (500 MHz, $CDCl_3$) δ 6.27 (s, 2H), 4.47 – 4.27 (m, 1H), 4.00 (dddd, J = 17.4, 12.0, 8.2, 2.9, 1H), 3.65 (s, 5H), 3.64 (s, 55H), 3.55 –3.53 (m, 3H), 3.37 (s, 3H), 2.64–2.57 (m, 2H), 1.51–1.41 (m, 2H), 1.32–1.26 (m, 2H), 1.16–1.09 (m, 2H), 1.08–1.01 (m, 2H).

Polymer P1 – ROMP copolymerization of 17 and 18. To a 2 mL scintillation vial were added 113 mg (0.312 mmol) of **17**. The vial was placed under vacuum and purged with nitrogen gas. To a separate 2 mL scintillation vial were added 11.06 mg (12.5 µmol) of third-generation Grubbs catalyst and 0.2 mL of dry DCM. Another 2 mL scintillation vial contained 372 mg of **18** and 0.2 mL of dry DCM. Both vials were placed under vacuum and purged with nitrogen gas. The solutions of catalyst and **8** were then injected into the reaction vial which was placed under vacuum. The reaction was stirred under nitrogen for 24 h. An aliquot of butyl vinyl ether was added to terminate the reaction. The polymer was precipitated in hexanes and placed under vacuum to afford a brown liquid. M_n = 138 kDa and *Đ* = 1.29 determined by SEC in THF. The **17**:**18** ratio was 43:57 as determined by ^{1}H NMR (see paper and Figure S12 for details).

pH Studies. Solutions of 30% (v/v) MeCN:H_2O PBS buffer were prepared. The buffer was prepared based on standard recipes for 1X pH 7.4 or 6.5 PBS buffer and fresh solution was made for each trial. The pH was adjusted to either 6.5 or 7.4 using dilute aqueous solutions of HCl and NaOH. Appropriate compound was weighed out for a final concentration of 10 mM, dissolved in a minimum amount MeCN, and added to the buffered solution. pH time points were taken every 3 s over 30 min.

NMR Studies. Solutions of 30% MeCN-d_3/D_2O PBS buffer were prepared. The buffer was prepared based on standard recipes for 1X pH 7.4 or 6.5 PBS buffer and fresh solution was made for each trial. The pH was adjusted to either 6.5 or 7.4 using dilute NaOD and DCl solutions in D_2O. The pD values of solutions were determined by adding a constant of 0.4 to the pH. NMR tubes were prepared with solution then locked

and shimmed to D_2O on the NMR. Appropriate volume of a solution of compound dissolved in MeCN-d_3 was added to the NMR tube for a final concentration of 10 mM and a final volume of 700 uL. After shimming once more, spectra were continuously recorded for 30 minutes. Each measurement was 16 scans.

LC-MS. A solution of 30% (v/v) MeCN:H_2O PBS buffer was prepared. The buffer was prepared based on standard recipes for 1X pH 7.4 or 6.5 PBS buffer. The pH was adjusted to 7.4 using dilute aqueous solutions of HCl and NaOH. Compound **5** was weighed out for a final concentration of 10mM, dissolved in a minimum amount MeCN, and added to the buffered solution. The solution was stirred for 30 min. After completion, the degradation byproducts were extracted with EtOAc (2 x 10 mL). The organics were combined, dried with Na_2SO_4, and concentrated by rotary evaporation. The obtained solid was submitted to LC-MS. Mobile phase A (v/v): 95% H_2O, 5% MeCN + 0.1% FA. Mobile phase B: 5% H_2O, 95% MeCN + 0.1% FA. Gradient: 100% A to 100% B over 8 min to 100% A over 2 min with a flow rate of 0.5 mL/min.

HPLC. A solution of 30% (v/v) MeCN:H_2O PBS buffer was prepared. The buffer was prepared based on standard recipes for 1X pH 7.4 or 6.5 PBS buffer. The pH was adjusted to 7.4 using dilute aqueous solutions of HCl and NaOH. Compound **5** was weighed out for a final concentration of 10mM, dissolved in a minimum amount MeCN, and added to the buffered solution. The solution was stirred for 30 minutes. After completion, the degradation byproducts were extracted with EtOAc (2 x 10 mL). The organics were combined, dried with Na_2SO_4, and concentrated under reduced pressure. The obtained solid was dissolved in MeCN. HPLC gradient acetonitrile in water, 0.1% TFA; 0%-100% over 35 min. The second fraction at 24.3 min was concentrated under reduced pressure followed by ^1H and ^{13}C NMR in $CDCl_3$.

^1H NMR spectra of 5 and 8-11

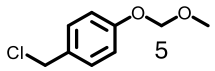

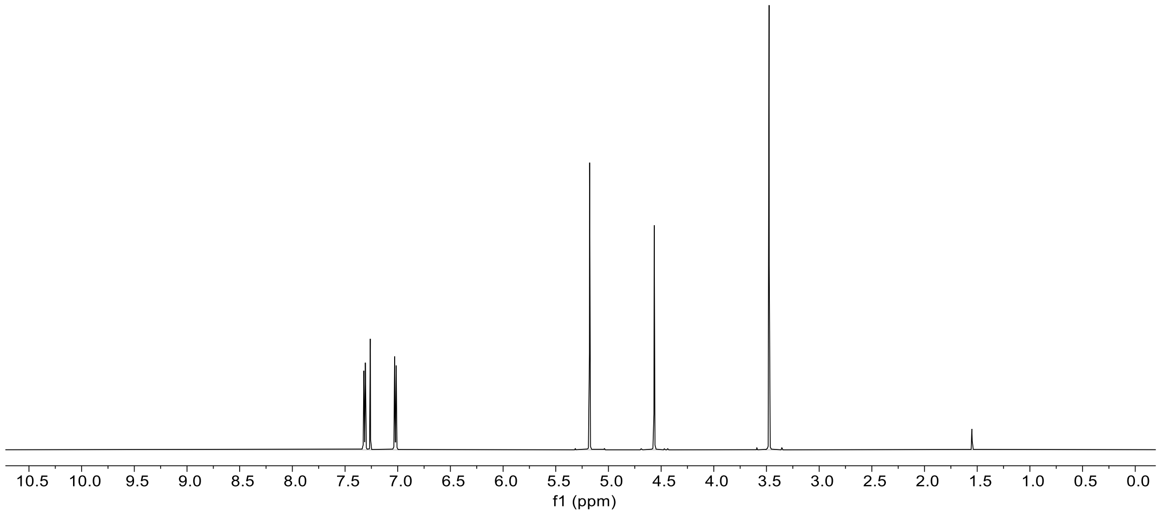

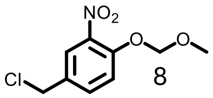

NO2
O
O
Cl

8

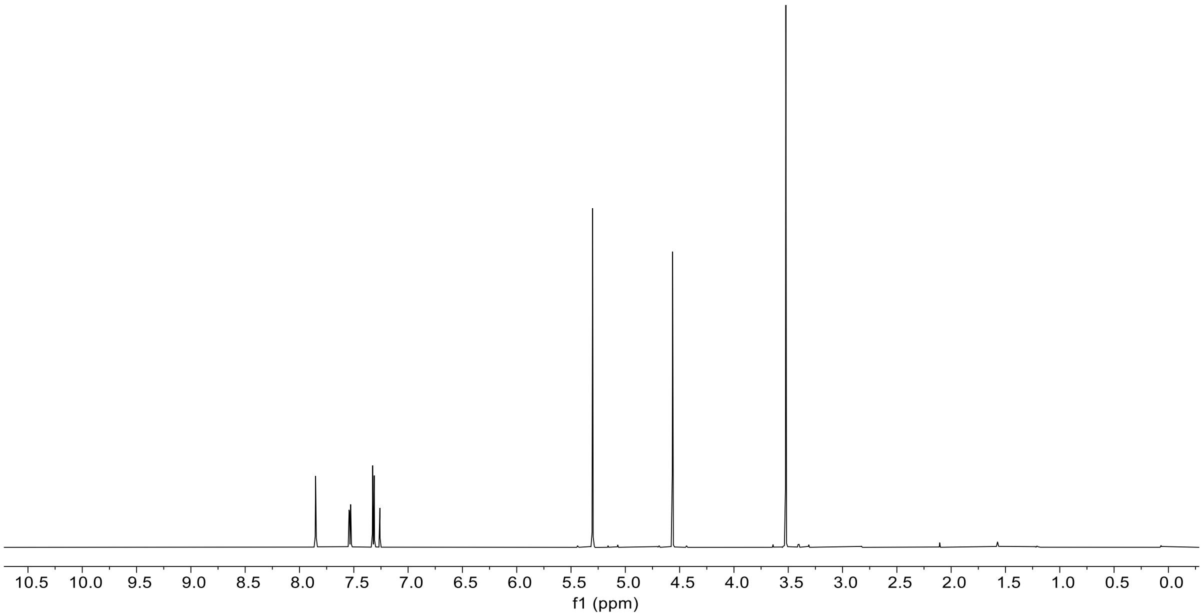

10.5 10.0 9.5 9.0 8.5 8.0 7.5 7.0 6.5 6.0 5.5 5.0 4.5 4.0 3.5 3.0 2.5 2.0 1.5 1.0 0.5 0.0
f1 (ppm)

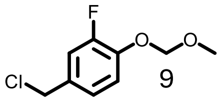
F
O
O
Cl

9

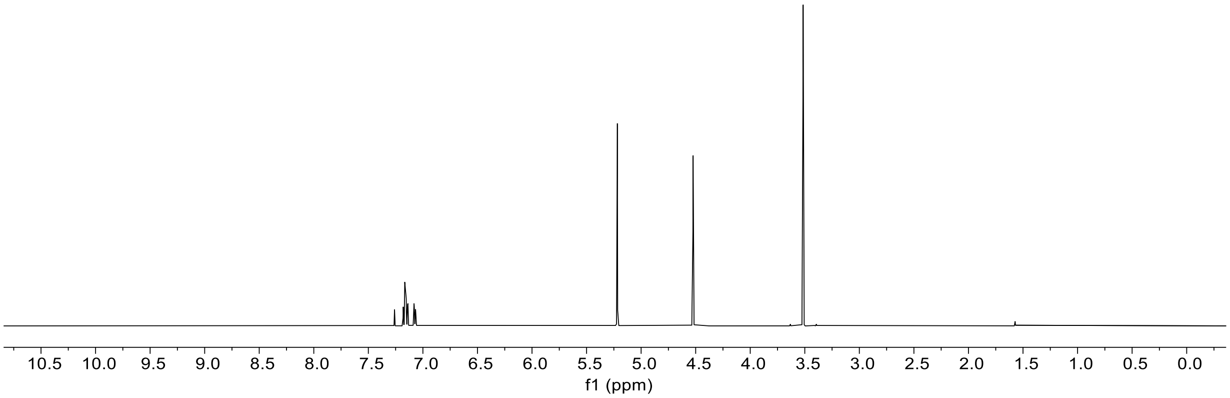
10.5 10.0 9.5 9.0 8.5 8.0 7.5 7.0 6.5 6.0 5.5 5.0 4.5 4.0 3.5 3.0 2.5 2.0 1.5 1.0 0.5 0.0
f1 (ppm)

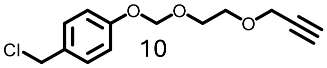
O
O
O
Cl

10

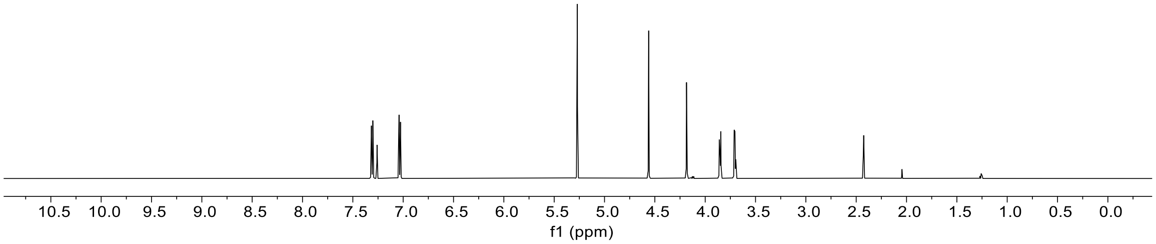
10.5 10.0 9.5 9.0 8.5 8.0 7.5 7.0 6.5 6.0 5.5 5.0 4.5 4.0 3.5 3.0 2.5 2.0 1.5 1.0 0.5 0.0
f1 (ppm)

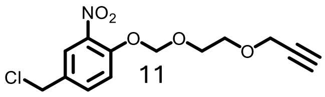

11

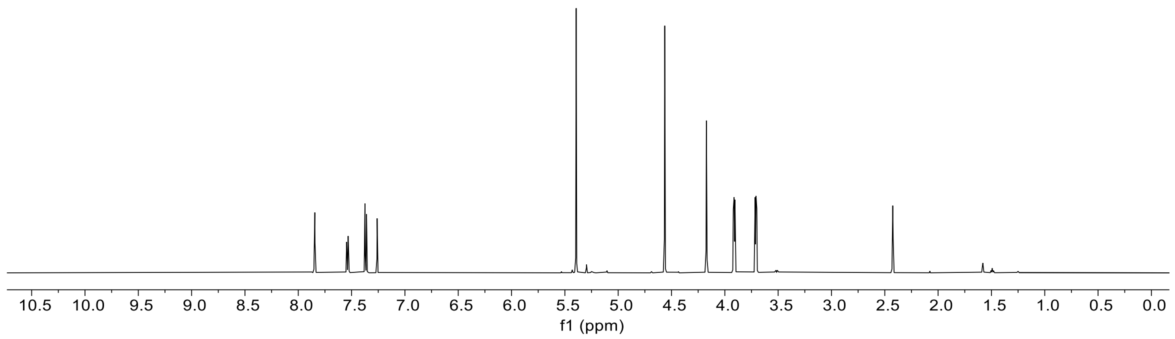

Additional Supporting Figures

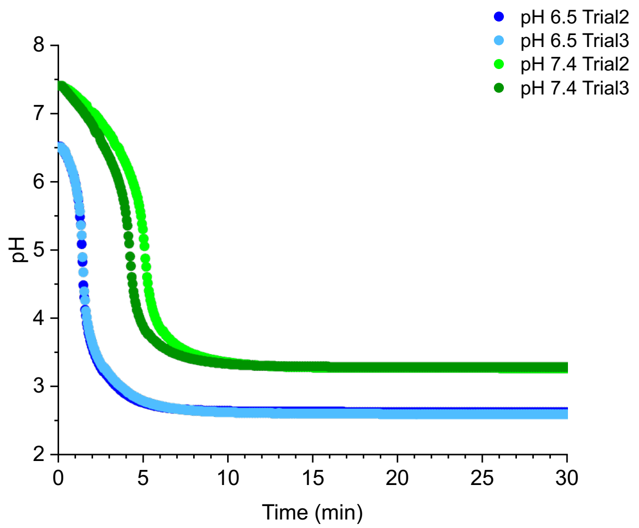

Figure S1. Additional pH trials of **5** in 30% (v/v) MeCN/H_2O PBS buffer. Reported errors are standard error over 3 trials.

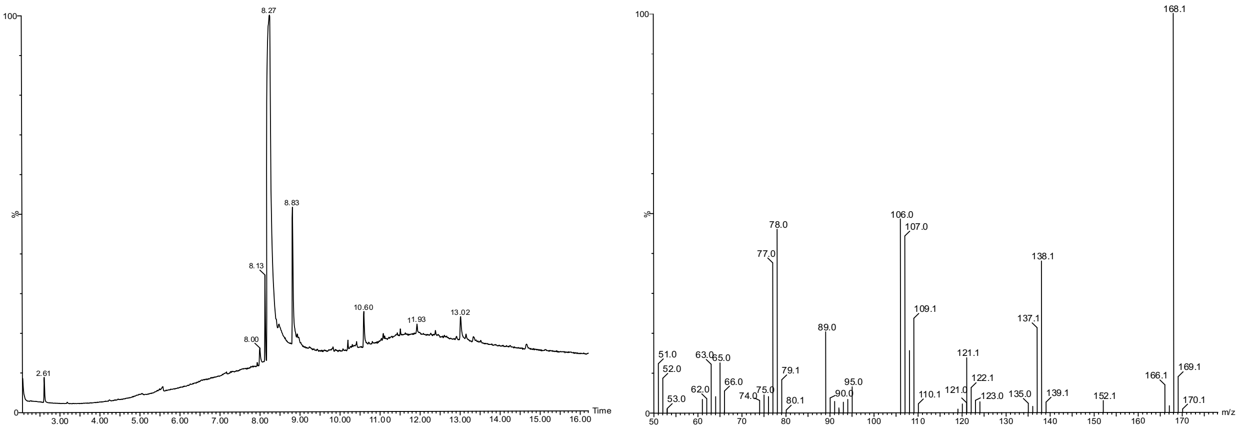

Figure S2. LC-MS of degradation products of **5**.

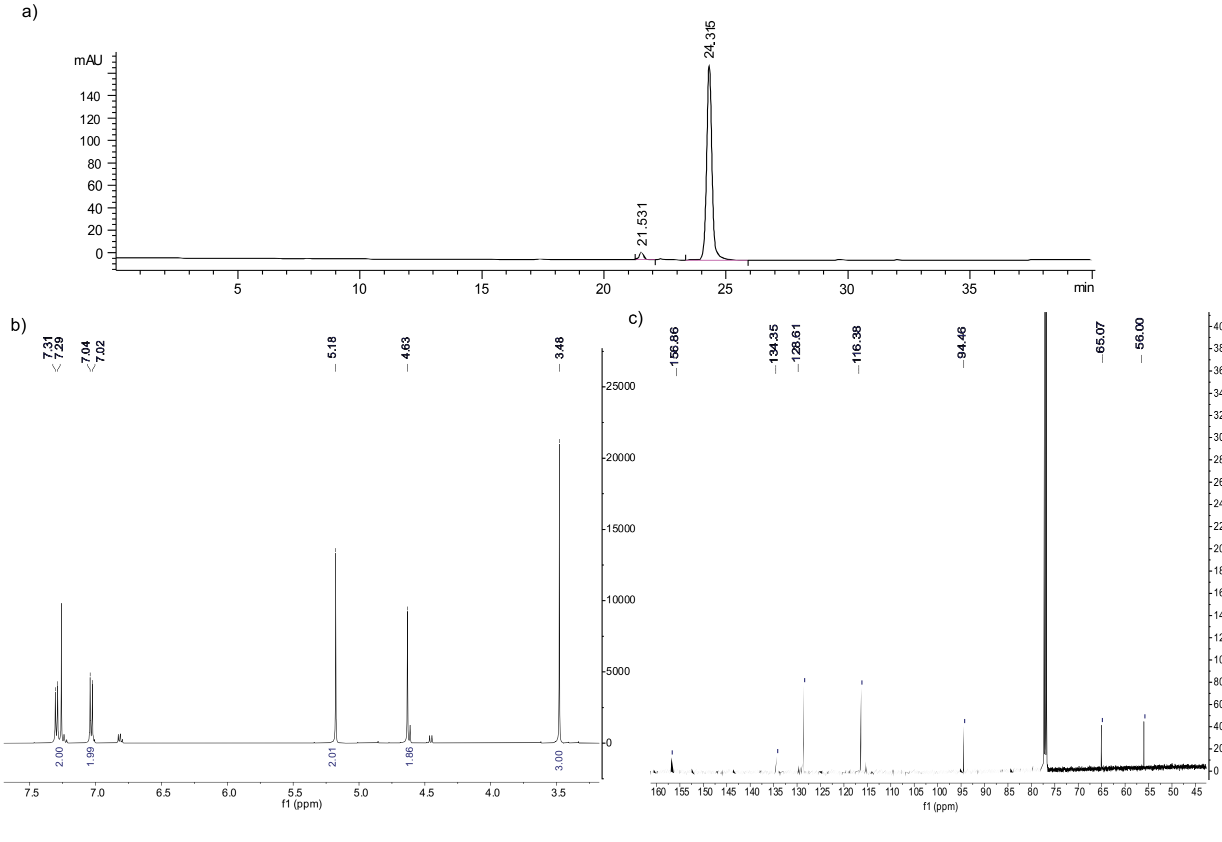

Figure S3. HPLC and NMRs of degradation products of **5**. (a) preparative HPLC plot. (b) ^{1}H NMR in CDCl3 from collected fraction at 24.3 min. (c) ^{13}C NMR in $CDCl_3$ from collected fraction at 24.3 min.

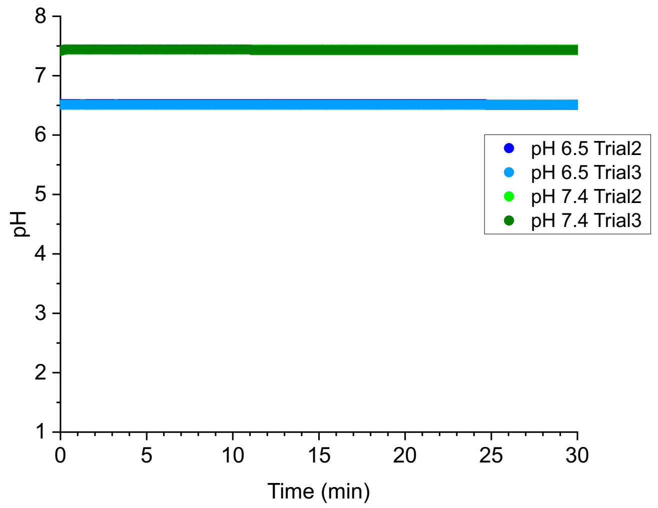

Figure S4. Additional pH trials of **8** in 30% (v/v) MeCN/H_2O PBS buffer. Reported errors are standard are standard error over 3 trials.

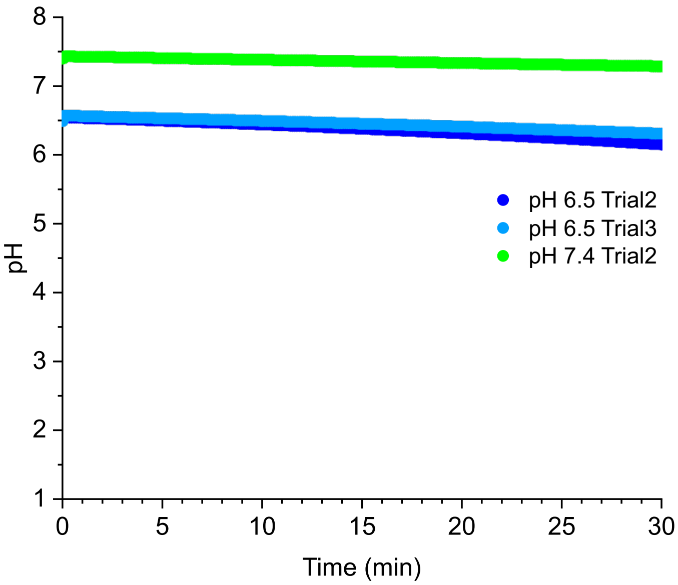

Figure S5. Additional pH trials of **9** in 30% (v/v) MeCN/H_2O PBS buffer. Reported errors are standard are standard error over 3 trials.

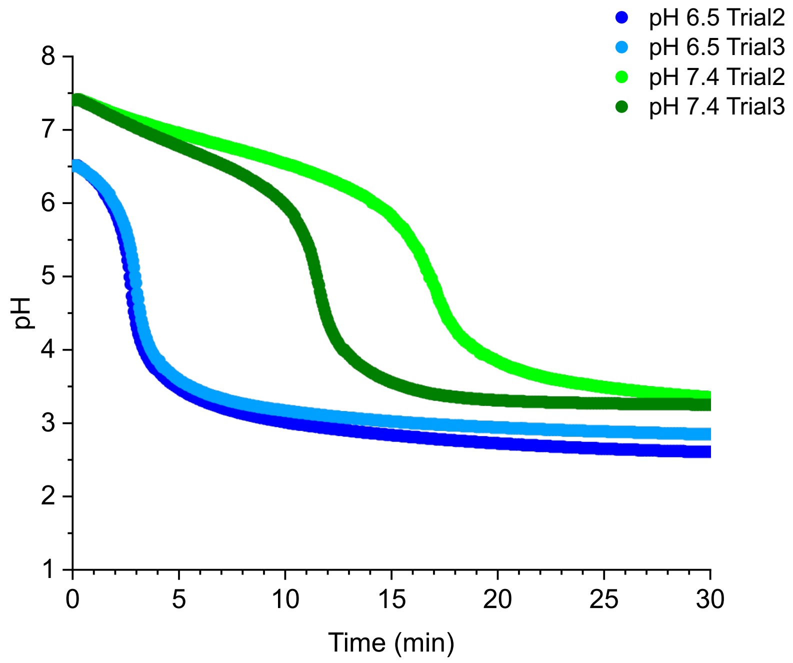

Figure S6. Additional pH trials of **10** in 30% (v/v) MeCN/H_2O PBS buffer. Reported errors are standard are standard error over 3 trials.

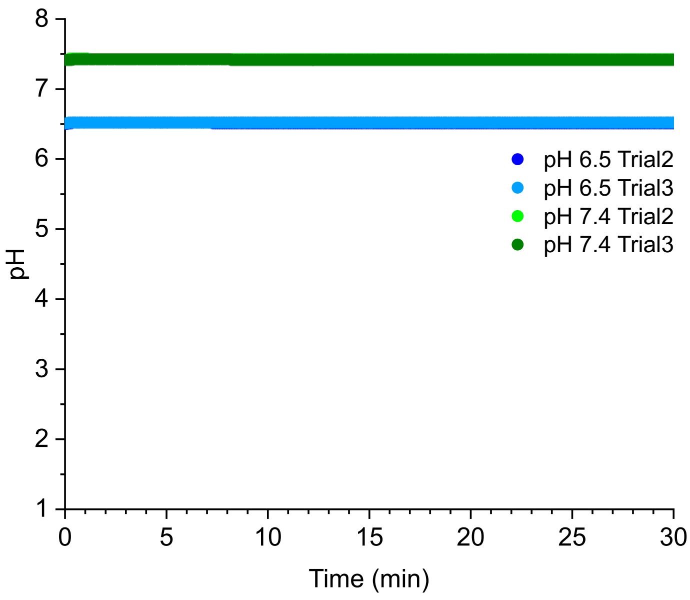

Figure S7. Additional pH trials of **11** in 30% (v/v) MeCN/H_2O PBS buffer. Reported errors are standard are standard error over 3 trials.

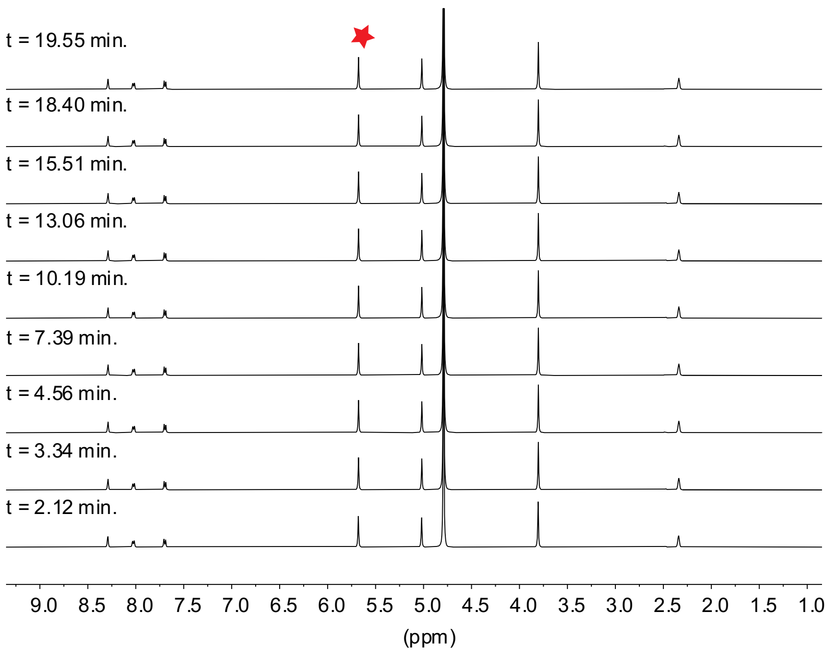

Figure S8. 1H NMR degradation of **8** in 30% (v/v) MeCN-d_3/D_2O PBS buffer at pD 7.4. The red star indicates the key acetal peak.

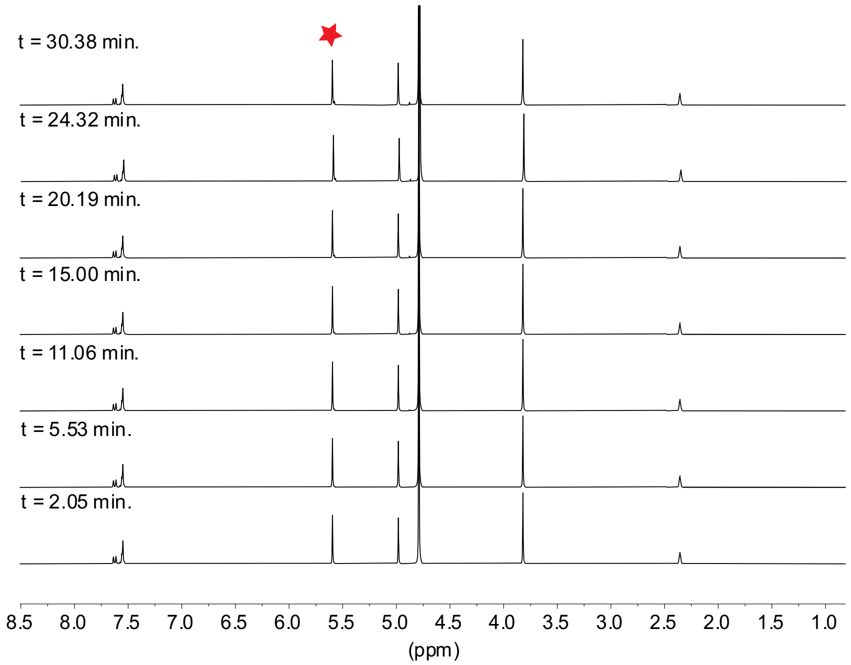

Figure S9. NMR degradation of **9** in 30% (v/v) MeCN-d_3/D_2O PBS buffer at pD 7.4. The red star indicates the key acetal peak.

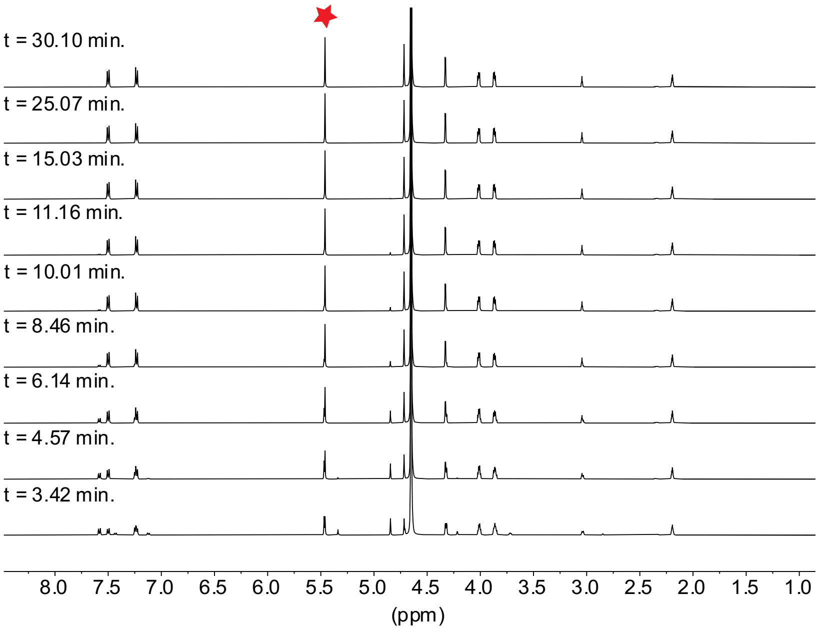

Figure S10. NMR degradation of **10** in 30% (v/v) MeCN-d_3/D_2O PBS buffer at pD 7.4. The red star indicates the key acetal peak.

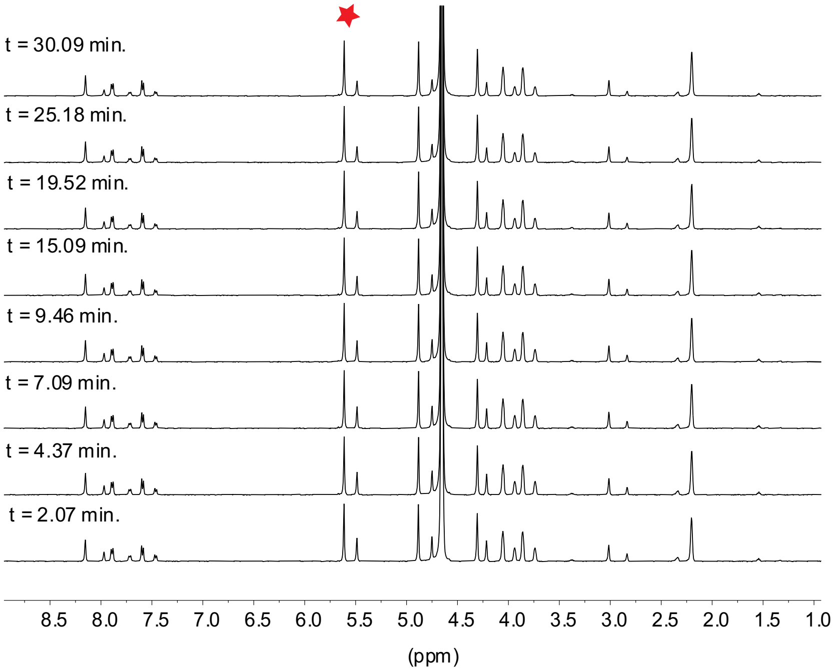

Figure S11. NMR degradation of **11** in 30% (v/v) MeCN-d_3/D_2O PBS buffer at pD 7.4. The red star indicates the key acetal peak.

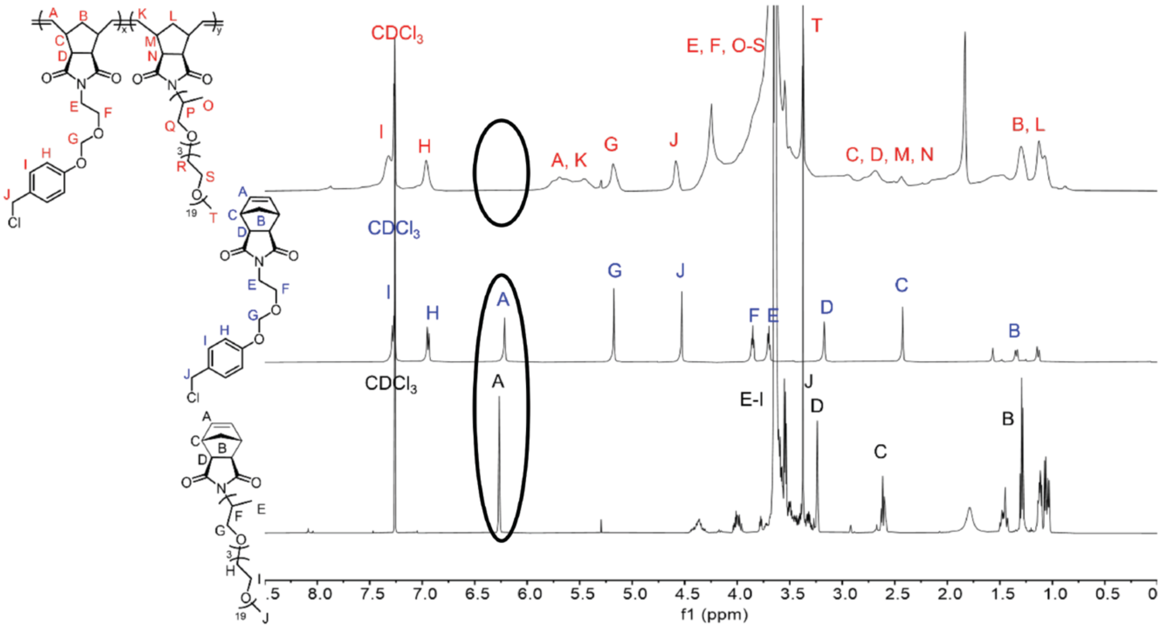

Figure S12. ROMP copolymerization of **17** and **18** evidenced by ^{1}H NMR. Black circles indicate the disappearance of norbornene double bonds, suggesting successful copolymerization.

References

(1) Sanford, M. S.; Love, J. A.; Grubbs, R. H. A Versatile Precursor for the Synthesis of New Ruthenium Olefin Metathesis Catalysts. *Organometallics* **2001**, *20*, 5314–5318.

(2) Choi, T. L.; Grubbs, R. H. Controlled Living Ring-Opening-Metathesis Polymerization by a Fast-Initiating Ruthenium Catalyst. *Angew. Chem. Int. Ed.* **2003**, *42*, 1743–1746.
